# Single-Cell Profiling Identifies Key Pathways Expressed by iPSCs Cultured in Different Commercial Media

**DOI:** 10.1016/j.isci.2018.08.016

**Published:** 2018-08-23

**Authors:** Maciej Daniszewski, Quan Nguyen, Hun S. Chy, Vikrant Singh, Duncan E. Crombie, Tejal Kulkarni, Helena H. Liang, Priyadharshini Sivakumaran, Grace E. Lidgerwood, Damián Hernández, Alison Conquest, Louise A. Rooney, Sophie Chevalier, Stacey B. Andersen, Anne Senabouth, James C. Vickers, David A. Mackey, Jamie E. Craig, Andrew L. Laslett, Alex W. Hewitt, Joseph E. Powell, Alice Pébay

**Affiliations:** 1 Centre for Eye Research Australia, Royal Victorian Eye and Ear Hospital, University of Melbourne, 32 Gisborne Street, East Melbourne, VIC 3002, Australia; 2 Ophthalmology, Department of Surgery, the University of Melbourne, Melbourne, VIC 3002, Australia; 3 Institute for Molecular Bioscience, University of Queensland, Brisbane, QLD 4072, Australia; 4 Commonwealth Scientific and Industrial Research Organisation (CSIRO) Manufacturing, Clayton, VIC 3168, Australia; 5 Australian Regenerative Medicine Institute, Monash University, Clayton, VIC 3168, Australia; 6 School of Medicine, Menzies Institute for Medical Research, University of Tasmania, Hobart, TAS 7000, Australia; 7 Wicking Dementia Research and Education Centre, University of Tasmania, Hobart, TAS 7000, Australia; 8 Centre for Ophthalmology and Visual Science, Lions Eye Institute, University of Western Australia, Perth, WA 6009, Australia; 9 Flinders University, Adelaide, SA 5042, Australia; 10 Garvan Institute of Medical Research, Darlinghurst, Sydney, NSW 2010, Australia

**Keywords:** Stem Cells Research, Transcriptomics, Automation

## Abstract

We assessed the pluripotency of human induced pluripotent stem cells (iPSCs) maintained on an automated platform using StemFlex and TeSR-E8 media. Analysis of transcriptome of single cells revealed similar expression of core pluripotency genes, as well as genes associated with naive and primed states of pluripotency. Analysis of individual cells from four samples consisting of two different iPSC lines each grown in the two culture media revealed a shared subpopulation structure with three main subpopulations different in pluripotency states. By implementing a machine learning approach, we estimated that most cells within each subpopulation are very similar between all four samples. The single-cell RNA sequencing analysis of iPSC lines grown in both media reports the molecular signature in StemFlex medium and how it compares to that observed in the TeSR-E8 medium.

## Highlights

• HiPSCs maintained in StemFlex and E8 were compared by single-cell transcriptomics
• Data revealed similar expression of core pluripotency genes
• Data revealed a shared three subpopulation structure different in pluripotency states

## Graphical Abstract

**Figure:**
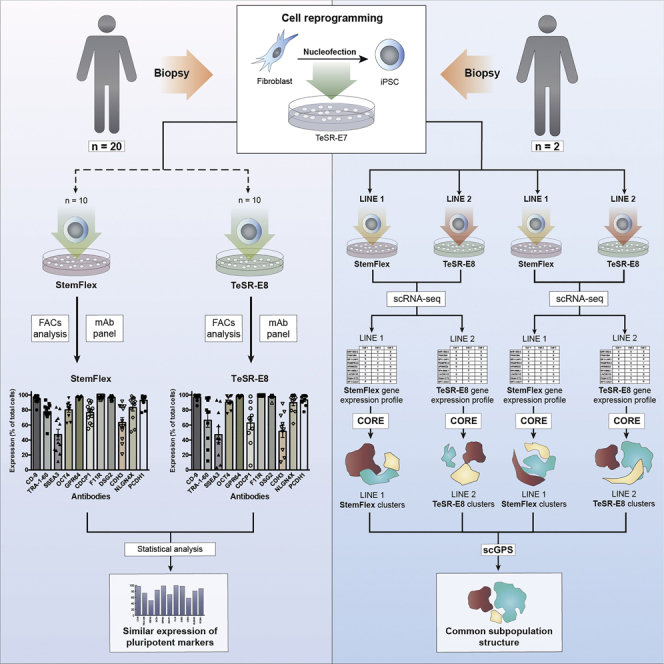

## Introduction

Somatic cells can be reprogrammed into induced pluripotent stem cells (iPSCs) using a combination of transcription factors involved in the maintenance of pluripotency (Park et al., 2007, Takahashi and Yamanaka, 2006, Takahashi et al., 2007, Yu et al., 2007). Like human embryonic stem cells (hESCs), human iPSCs provide a powerful means by which to investigate the pathogenesis of disease because these cells can be differentiated into relevant cell types of interest for disease modeling, drug screening, and delving into the fundamental aspects of disease pathology, as well as for regenerative medicine. Numerous protocols have been described for the maintenance of human pluripotent stem cells, using different matrices and culture media (International Stem Cell Initiative Consortium et al., 2010). The original method of maintenance of hESCs was based on a feeder layer of embryonic fibroblasts and a serum-based medium (Reubinoff et al., 2000, Thomson, 1998). It evolved to more defined conditions using serum-free media with key signaling molecules (Furue et al., 2008, Pébay et al., 2005, Vallier et al., 2005, Xu et al., 2005) and/or feeder-free conditions (including the use of Matrigel, collagen, vitronectin, or laminin) (Klimanskaya et al., 2005, Ludwig et al., 2006). Various commercialized serum-free media have now become available with an ongoing evolution to fully defined media, from KnockOut Serum Replacement medium supplemented with basic fibroblast growth factor to the TeSR family media (mTeSR1, TeSR2, and TeSR-E8) (Chen et al., 2011, Ludwig et al., 2006, Sun et al., 2009), and the newly released StemFlex, a medium with proprietary compounds, and hence of unknown formulation and with robustness efficiency remaining to be characterized.

Although there have been clear advances in the maintenance, standardization, and upscaling of pluripotent stem cell culture, some limitations remain and need to be addressed to realize the translational potential of iPSCs (Kim and Kino-oka, 2018). Indeed, human variability is a main source of differences observed between cell lines generated and maintained in various laboratories (Allegrucci and Young, 2006, Allegrucci et al., 2007), with genetic background and methodologies also having a significant contribution (Kilpinen et al., 2016). The automation of pluripotent stem cell culture provides an efficient way to improve these aspects, increasing throughput and standardizing many aspects of cell culture, including in iPSC generation, maintenance, passaging, and differentiation into progeny cells of interest (Crombie et al., 2017, Konagaya et al., 2015, Paull et al., 2015). These automated steps allow minimal variation in each procedure, reduce inter-sample variability, and hence increase robustness of cell culture procedures (Daniszewski et al., 2017).

Using an automated platform to ensure robustness of cell culture, we compared the cellular and molecular profiles of human iPSCs maintained in a standard TeSR-E8 medium and in the newly released StemFlex. Quality control was assessed by markers of pluripotency, three germ layer differentiation, and virtual karyotyping. We then compared samples grown in both media by single-cell RNA sequencing (scRNA-seq) to uncover the molecular signature underlying pluripotency, and report on whether similar pathways are modulated in both conditions.

## Results

**Figure 1:**
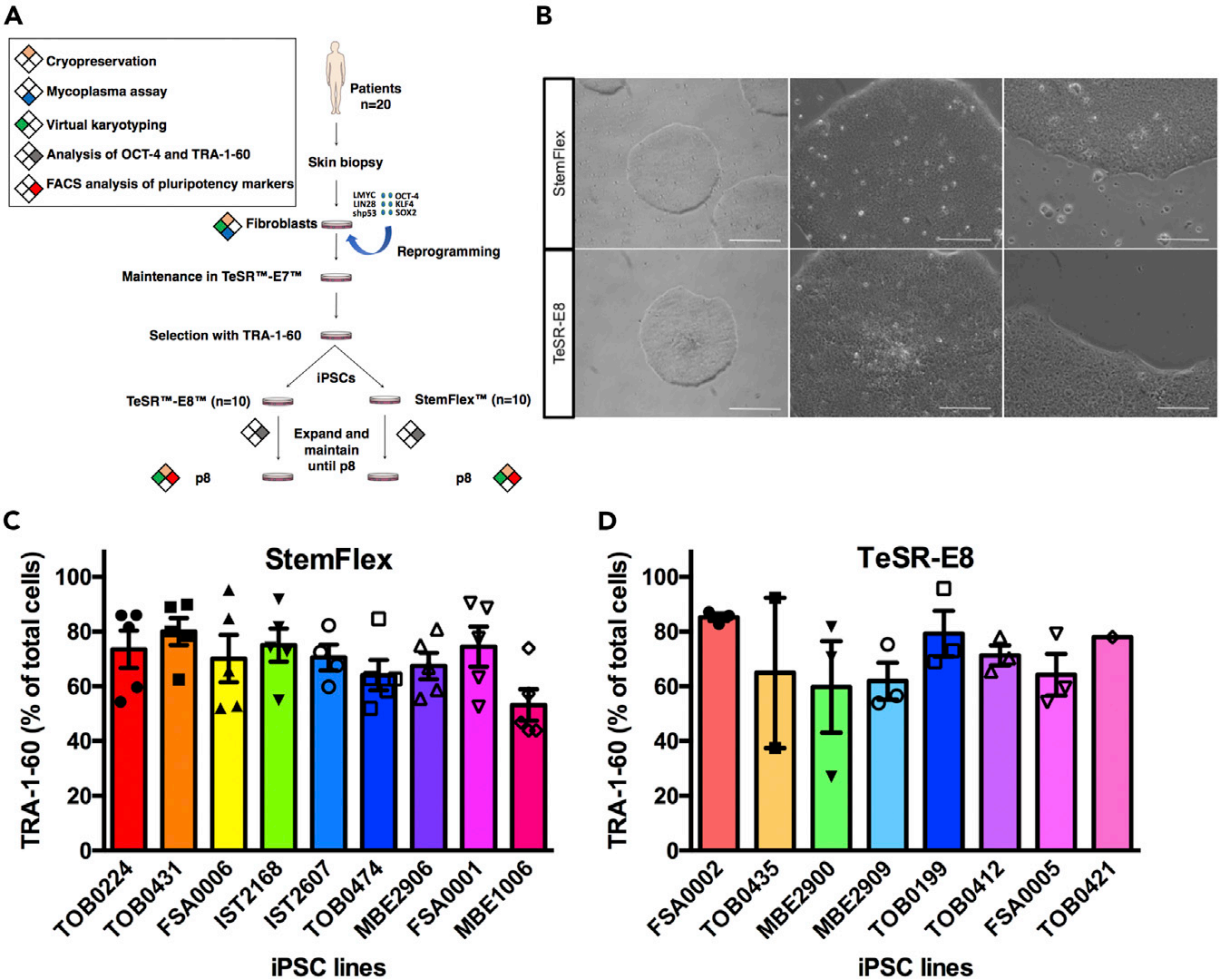
Quality Control of iPSCs Cultivated in StemFlex and TeSR-E8 (A) Schematic representation of the experimental plan. (B) Representative morphology of iPSCs maintained in StemFlex or TeSR-E8 post-passaging. Images of human iPSCs, at day 8 post-passaging, at different magnifications (×4, ×10, and ×20). Cells were cultivated using an automated platform, on vitronectin, in StemFlex (IST2607, p3 1:6 ratio) or TeSR-E8 (TOB0435, p2 1:3 ratio). Images are representative of all cell lines. Scale bars: 100 μM. (C and D) Quantification of TRA-1-60-positive cells before passaging of human iPSCs maintained in StemFlex (C) or TeSR-E8 (D). Scatterplot with bar of the TRA-1-60-positive (+ve) cells just before passaging, in independent iPSC lines grown in StemFlex (TOB0224 p3,5–8; TOB0431 p3,5–8; FSA0006 p3,5–8; IST2168 p3,5–8; IST2607 p3,5,6,8; TOB0474 p3,5–8; MBE2906 p3,5–8; FSA0001 p2,4–6,8; MBE1006 p2,4–6,8) or TeSR-E8 (FSA0002 p2,5,8; TOB0435 p5,8; MBE2900 p2,5,8; MBE2909 p4,5,8; TOB0199 p2,4,8; TOB0412 p3,4,8 FSA0005 p4,5,8 TOB0421 p8). The abnormal lines TOB0218 (StemFlex), MBE2899 (TeSR-E8), and MBE2901 (TeSR-E8) were not included in this analysis. Each column represents the mean ± SEM of successive passages of individual lines. Two-tailed t test indicates no statistical difference in TRA-1-60 expression between the two culture conditions (p = 0.8546).

**Figure 2:**
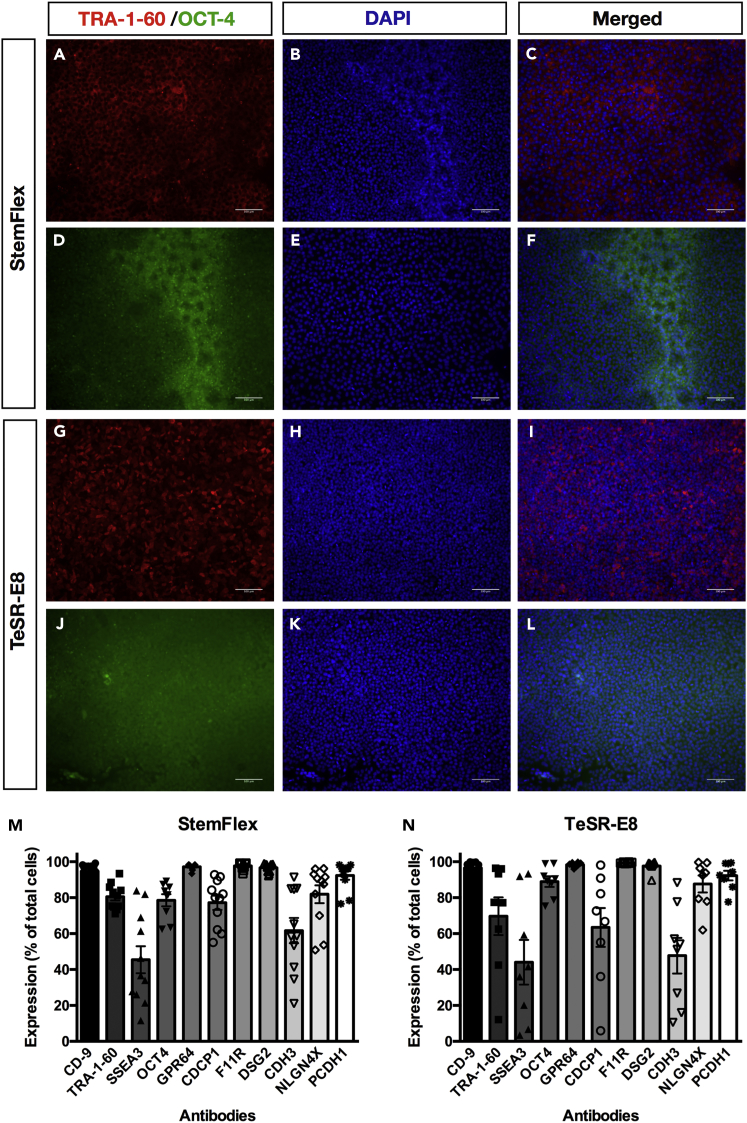
Expression of Pluripotency Markers of Human iPSCs Maintained in StemFlex or TeSR-E8 (A–L) Representative immunostainings for TRA-1-60 and OCT-4. Images of human iPSCs cultivated in StemFlex (TOB0474) or TeSR-E8 (MBE2901) at day 8 post-passaging (p8), for TRA-1-60 (A and G) or OCT-4 (D and J), with DAPI nuclei counterstain (B, E, H, and K) and merged images (C, F, I, and L). Images are representative of all cell lines. Scale bars: 100 μM. (M and N) Quantification of pluripotency markers. Scatterplot with bar of human iPSCs cultivated in (M) StemFlex (TOB0224; TOB0431; FSA0006; IST2168; IST2607; TOB0474; MBE2906; FSA0001; MBE1006) or (N) TeSR-E8 (TOB0421; FSA0005; TOB0199; FSA0002; MBE2909; TOB0435; MBE2900; TOB0412) at day 8 post-passaging (p16), for CD9, TRA-1-60, SSEA-3, OCT-4, GPR64, CDCP1, F11R, DSG2, CDH3, NLGN4X, and PCDH1. Each column represents the mean ± SEM of 10 individual lines. FSA0006 and TOB0474 were analyzed twice for all markers aside from OCT-4, which was analyzed once. The abnormal lines TOB0218 (StemFlex), MBE2899 (TeSR-E8), and MBE2901 (TeSR-E8) were not included in this analysis. Two-way ANOVA followed by Sidak's multiple comparisons test indicates no statistical difference in each pluripotency marker expression between the two culture conditions.

Donor iPSCs were derived as described in Crombie et al. (2017). Following TRA-1-60 selection, 10 lines were maintained in StemFlex medium and 10 lines in TeSR-E8 medium (Figure 1A). We had already adapted automation for the culture of pluripotent stem cells using TeSR-E8 (Crombie et al., 2017), and StemFlex also allowed maintenance on the automated platform, with colonies showing typical pluripotent stem cell morphology (Figures 1B and S1) and similar levels of expression of TRA-1-60 (Figures 1C and 1D). After 8 passages, all iPSC lines for both media expressed the pluripotency markers OCT-4 and TRA-1-60 (Figures 2A–2L). All iPSC lines in both conditions were able to differentiate to the three germ layers, as assessed by embryoid body formation (Figure S2). Virtual karyotyping using SNP analysis revealed post-reprogramming anomalies in two lines grown in TeSR-E8 (MBE2899, MBE2901) and one line maintained in StemFlex (TOB0218) (Figure S3). At passage 16, iPSCs grown in both media were subjected to flow cytometry for a panel of monoclonal antibodies that detect human pluripotent stem cells (O'Brien et al., 2017), increasing the stringency of the characterization of cell pluripotency markers. Cells retained high levels of expression of the panel of pluripotent markers in both media (Figures 2M and 2N). Of note, SSEA-3 was found to be low in all samples; however, this marker is known not to be essential to pluripotency (Brimble et al., 2007).

**Figure 3:**
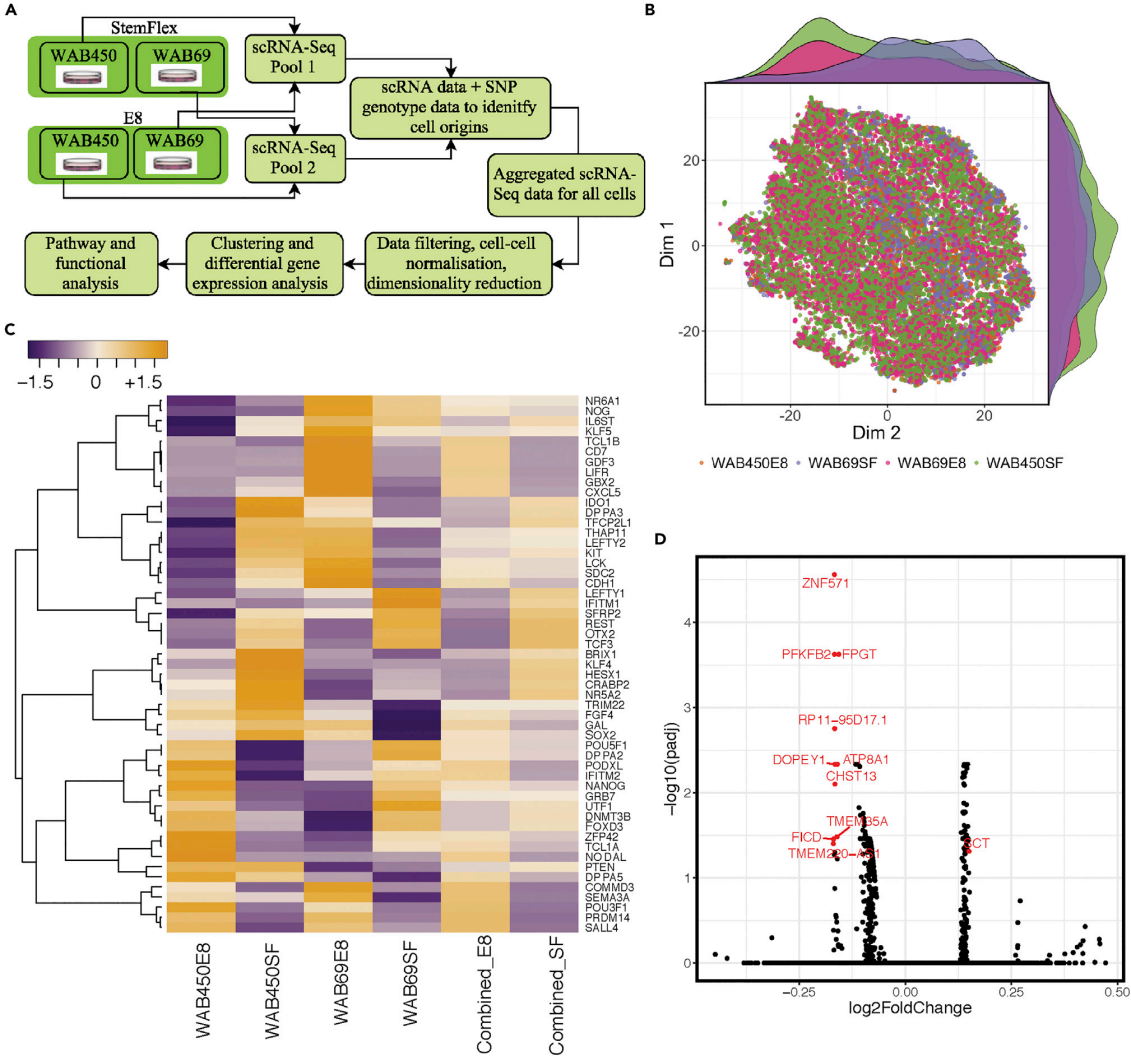
Comparing Effects of the Two Media at Single-Cell Level (A) Schematic representation of the experimental randomization design. Two cell lines were cultured separately in two media. The four cell cultures were genotyped (HumanCore Beadchip arrays) and were randomly combined into two pools for scRNA-seq experiment (10X Chromium). Cells were assigned to original sample based on sample SNP genotype and single-cell SNPs called from scRNA-seq data. The scRNA-seq data were aggregated for all four samples, filtered, normalized, clustered, and analyzed for differential gene expression and functional pathways. (B) Two-dimensional distribution of cells based on gene expression profile. Dimensions 1 and 2 are principal coordinates of imputed values from CIDR (Clustering through Imputation and Dimensionality Reduction) (Lin et al., 2017). The overlap between the four culturing samples suggests the overall similar effects of the two media. (C) Heatmap analysis of known pluripotency markers shown for four separate samples or the combination of two samples. Standardized gene expression is shown from low (purple color) to high (orange color). The numbers of genes expressed highly in StemFlex or in TeSR-E8 are similar, suggesting the two media maintain similar pluripotency states. (D) Volcano plot shows genome-wide differential expression results between cells in StemFlex versus cells in TeSR-E8 (positive log2Folchange indicates higher expression in StemFlex). The numbers of upregulated genes in each media are similar. Red text indicates genes for which upregulation is statistically significant between the two conditions.

We interrogated the molecular pathways involved in StemFlex and TeSR-E8 maintenance of pluripotency. We reprogrammed two independent donor fibroblast lines, WAB0450 and WAB0069; selected iPSCs using TRA-1-60 beads; and separated each iPSC line into the two media post-passage 1 (Figure 3A). Following virtual karyotyping at passage 8 to ensure that cells did not acquire chromosomal abnormalities (Figures S4A–S4F), and for confirmation of pluripotency by embryoid body formation (Figure S2), iPSCs were harvested for scRNA-seq to identify potential variation in pluripotency, associated pathways, and potential culture medium signature. Passage 8 was chosen because the number of aberrant cells with aberrant copy number variation (CNVs) is already reduced from early passages (Hussein et al., 2011). At day 5 post-passaging, iPSCs were dissociated into single cells, and live cells were selected with propidium iodide by fluorescence-activated cell sorting (FACS). Live cells were used for library preparation and scRNA-seq, as outlined in Figure 2A. Processing of our initial analysis identified a total of 21,597 cells in four samples. We filtered a total of 635 cells, which met one or more of the following criteria: high/low mapped reads (163 cells), high mitochondrial expression (456 cells), and high ribosomal expression (57 cells), resulting in 20,962 cells remaining for subsequent analysis. We mapped reads to a reference transcriptome (GRCh38p10) containing 32,838 genes, of which we filtered 16,459 because they were detected in less than 0.1% of the total number of cells leaving 16,379 genes for the analysis. The filtered data were normalized, and cells were then divided into individual samples and clustered separately. The distribution of cells based on gene expression profile showed a clear overlap between the four culturing samples, suggesting the overall similar effects of the two media (Figure 3B). For identifying genes and pathways involved in maintaining pluripotency, we performed differential gene expression (DE) between media, assessing both individual lines and pooled lines: WAB0450_E8 was analyzed together with WAB0069_E8 and WAB0450_SF together with WAB0069_SF followed by analysis of individual lines (Table S1). Furthermore, we performed both targeted expression analysis of 53 known pluripotency markers (Figure 3C) and genome-wide expression comparisons for all 16,379 genes (Figure 3D) in the four separate samples or in the pooled media. Gene expression was compared both by expression values (Figures 3C and 3D) and by percent of expressing cells (Tables S1 and S2, Figure S5). Cell cultures retained expression of the panel of pluripotent cell surface markers (Table S2). Notably, 99.96% of all cells in TeSR-E8 and 99.97% of all cells in StemFlex expressed at least one of the three core pluripotency markers, namely, *POU5F1*, *SOX2,* and *NANOG*, suggesting that cells are maintained in a pluripotency state (Table S2, Figure S5). The percentage of cells expressing *NANOG* is at 75.5^th^ percentile of all 16,270 reliably detected genes, higher than 13,349 genes. The distribution of number of detected cells for every gene suggests that *NANOG* was detected in more cells than most other genes (Figure S6). Although the total number of up- and downregulated genes in cells in the two media are similar (Figures 3C and 3D), a number of differentially regulated genes were unique for cells in each medium (Table S1). Pluripotency markers expressed higher in cells maintained in StemFlex include *LEFTY1*, *LEFTY2*, *IFITM1*, *SFRP2*, *REST*, *OTX2*, *TCF3*, *BRIX1*, *KLF4*, *KLF5*, *HESX1*, *CRABP2*, *NR5A2*, *FOXD3*, *DNMR3B*, *UTF1*, and *PTEN* (Figure 3C). These genes are particularly enriched for “Signaling by NODAL” (Reactome pathway analysis, false discovery rate [FDR] < 4 × 10^−3^). Gene markers expressed more in cells maintained in TeSR-E8 include *TCL1B*, *CD7*, *GDF3*, *LIFR*, *GBX2*, *CXCL5*, *CDH1*, *FGF4*, *GAL*, *SOX2*, *POU5F1*, *DPPA2*, *PODXL*, *IFITM2*, *NANOG*, *ZFP42*, *TCL1A*, *NODAL*, *DPPA5*, *COMMD3*, *SEMA3A*, *POU3F1*, *PRDM14*, and *SALL4* (Figure 3C). These TeSR-E8-upregulated genes are enriched for “*POU5F1*, *SOX2*, *NANOG* repress genes related to differentiation and activate genes related to proliferation” (Reactome pathway analysis, FDR < 1 × 10^−4^). Notably, both sets of upregulated genes share the main enriched pathway “Transcriptional regulation of pluripotent stem cells” (Reactome pathway analysis, FDR < 4 × 10^−3^ and FDR <1.9 × 10^−15^) (Figure 3C, Tables S1 and S2, Figure S7). We observed that the combination of 18 markers associated with the naive pluripotency (Collier et al., 2017, Guo and Smith, 2010, Ng and Surani, 2011, Warrier et al., 2017, Weinberger et al., 2016) were expressed by a low but comparable percentage (with difference lower than 1%) of cells grown in both conditions (Table S2). Moreover, genes driving transition from naive to primed pluripotency, i.e., *FGF4*, *FOXD3,* and *OTX2* (Collier et al., 2017) were also identified in subpopulations from all samples; however, on average the difference between the two media did not exceed 2% (Table S2). We also found high expression of genes involved in “Proliferation and survival” pathways and in “Metabolism” pathways (Table S2).

We then performed high-resolution comparisons at subpopulation and single-cell levels. Through clustering of the 20,962 cells, we consistently identified three distinct subpopulations in each of the WAB0450_SF, WAB0450_E8, WAB0069_E8, and WAB0069_SF samples (Figures S7–S9, Tables S4 and S5). The subpopulations were named 1, 2, and 3 based on the order of the total cell counts, with subpopulation 1 having the highest count. The dendrogram trees (Figures S7 and S8) show the clustering results, from applying our unbiased clustering method described previously (Nguyen et al., 2018). Statistical analysis of DE between the identified subpopulations, followed by pathway enrichment analysis of significantly regulated genes, suggests two major distinct pluripotency states. Subpopulations 1 and 2, which together account for 77%–95% of total cells in a sample (Figures S7–S9), had gene expression signature closer classifying them as more pluripotent cells compared with cells in subpopulation 3, which are more primed to differentiation (5%–23%, Tables S3 and S6). Table S3 shows enriched pathways for upregulated genes when comparing subpopulations 1 and 2 to subpopulation 3. The comparison was performed separately for each of the four samples. This comparison allows the independent assessment of the three subpopulations in each cell line and each medium. In addition, Table S6 shows functional pathway analysis for the common gene markers expressed highest in each of the three subpopulations and found consistently in all four samples. This comparison combines the shared pattern found in all four samples, thereby reinforcing the potential gene markers for each of the three subpopulations. Overall, the enrichment of pluripotency for subpopulations 1 and 2 compared with subpopulation 3 is consistent across cell lines and media (Tables S3 and S6), which is consistent with results from our previous study of a human iPSC population (Nguyen et al., 2018). Specifically, subpopulations 1 and 2 had a higher expression level of pluripotency markers, with significant enrichment (p adjusted < 0.001) of the Reactome pathway “Upregulation of transcriptional regulation of pluripotent stem cells” (>20 genes in the pathway were significantly upregulated, Table S3). For example, consistently in four samples (WAB0450_SF, WAS0450_E8, WAB0069_SF, and WAB0069_E8), the RNA levels of key pluripotency gene markers such as *POU5F1*, *SOX2,* and *NANOG* were higher in cells within subpopulations 1 and 2 than in cells within subpopulation 3 (Table S3). Notably, the percent of cells expressing these markers were higher in subpopulation 3 than in the remaining cells, possibly due to the relatively smaller number of cells present in this subpopulation (5%–23% of the total cells). Compared with subpopulations 1 and 2, the cells in subpopulation 3 were more primed to differentiation, with the significant upregulation (FDR <0.05) of differentiation-promoting pathways, such as “Signaling by NOTCH4” (FDR <3.6 × 10^−7^), “RUNX1 regulates transcription of genes involved in differentiation of HSCs” (FDR <1.5 × 10^−5^), “Beta-catenin-independent WNT signaling” (FDR <0.0003), and “TCF dependent signaling in response to WNT” (FDR <0.008, Table S6). The complete list of all gene markers that are expressed highest in each subpopulation are shown in Table S6.

To compare subpopulation structure between two independent samples (e.g., same cell line in two media, or different cell lines in two media), we applied two approaches. First, we performed unbiased clustering of cells in each of the sample and compared the clustering results between separate samples and between individual sample with the combined set of all cells from four samples (Figures S7–S10). The grouping of similar cells into clusters (subpopulations) reflects the structure of the cell population in the original population, because the clustering is data driven and does not require prior knowledge and assumptions on the number of clusters. We consistently found three subpopulations in each of the four samples, suggesting the shared subpopulation structure (Figures S7 and S8). We found that the distribution of cells into cell-cycle stages is similar between the four samples, suggesting that the subpopulation structure is not driven by cell-cycle phases (Figure S9). Moreover, we performed clustering analysis for the merged dataset of all cells from all four samples (2 cell lines × 2 media). We also found three subpopulations, and importantly that the percent of cells allocated to subpopulations 1, 2, and 3 are similar between all four samples, suggesting that the subpopulation structure is independent of cell lines and culturing media (Figures S7 and S8). Second, to quantitatively compare between subpopulations identified separately in each of the four samples, we performed a machine learning model, which estimates pairwise transition scores between subpopulations from one sample to another. For each pair of subpopulations identified through clustering, we run scGPS (single-cell global fate potential of subpopulation), which is a machine learning classification method to compare every cell from one subpopulation to another subpopulation, as described in Nguyen et al. (2017). This method was applied to quantitatively estimate conditional class probability of every single cell belonging to a subpopulation, thus allowing to estimate the percent of similar cells between two subpopulations. We found that approximately 98% of the cells from subpopulation 3 in one sample were similar to cells in subpopulation 3 from the other sample, much higher than the similarity when compared with cells in subpopulations 1 and 2 (Table S4). Thus, the analysis suggests that all samples share the common subpopulation 3. Similarly, subpopulations 1 of one cell line (WAB0069) or two different cell lines in StemFlex and TeSR-E8 media are 95.0% and 95.8% similar, suggesting subpopulations 1 are the same among these samples (Table S4). Notably, subpopulations 1 and 2 in the cell line WAB00450_E8 are less specific and have the potential to convert into subpopulations 1, 2, and 3. Interestingly, subpopulations 2 in WAB0450_E8 and WAB0069_E8 samples display an intermediate state and have a high (ranging from 82% to 100%) similarity score (i.e., high potential to be the same cell types/cell state) compared with subpopulations 2 or 3 in WB0069_SF (Table S4). Overall, we found a shared subpopulation structure among all samples, in which subpopulations 1 and 3 are more discrete and present in all samples, whereas subpopulation 2 is more transient and has the potential to dynamically transition between subpopulations 2 and 3.

## Discussion

We analyzed the ability of the recently released StemFlex and of TeSR-E8, a widely used cell medium, to maintain pluripotency of newly reprogrammed human iPSCs. We used an automated platform to perform this analysis to ensure that variations observed in cell maintenance were independent of human variability. All lines maintained in each medium (10 independent lines per media) demonstrated robust maintenance of pluripotency, with key colony morphology and expression of pluripotency markers, analyzed by immunochemistry and FACS analysis at passage 16. Most lines retained a normal karyotype with the exception of two in TeSR-E8 and of one in StemFlex. Our data thus support the robustness of both media to maintain iPSCs, as cells grown in both conditions display the morphological and molecular characteristics of iPSCs. In-depth molecular analysis was then performed using scRNA-seq to access the molecular network in place in cells grown in both culture conditions. Two independent fibroblast cultures were reprogrammed, and the two generated iPSC lines were then maintained and passaged in both media in parallel. Following virtual karyotyping, cells from different cell lines and media were then combined before single-cell sequencing. This study design was chosen to limit the inter-variability observed between independent iPSC lines. The analysis of gene expression profiles in the four samples—two lines each maintained in the two media—indicate that they share a very similar global gene expression signature, although specific pathways are enriched for cells in each of the two media. Our analysis identified that the core pluripotency factors, i.e., *OCT-4*, *NANOG,* and *SOX2,* were expressed by similar proportions of cells in both StemFlex and TeSR-E8 (Table S2), and >99.9% of all cells expressed at least one of these three factors, which is an indication of potency of both media to maintain cells in a pluripotent state, as levels of expression of those transcription factors are tightly correlated and critical to maintain pluripotency (Rizzino, 2009). At individual gene levels, we observed that on average 18.1% and 19.5% of TeSR-E8- and StemFlex-cultured cells, respectively, expressed *NANOG* (range 9.9%–44.5%; Tables S2 and S5). Its heterogeneous expression was reported previously both in mouse embryonic stem cells (Chambers et al., 2007) and human iPSCs (Yu et al., 2018), and it was suggested that *NANOG* levels in cells exist in a state of dynamic equilibrium (Boyer et al., 2005, Loh et al., 2006). The low levels of *NANOG* found in this study are consistent with previous results from an scRNA-seq iPSC dataset, showing *NANOG* expression level to be approximately 47 times lower than that of *OCT-4* (Nguyen et al., 2018). *OCT-4* is also essential for acquisition of naive pluripotency, and for differentiation (Radzisheuskaya et al., 2013). Furthermore, *OCT-4* and *SOX2* co-regulate their expression (Chew et al., 2005), while they jointly regulate *NANOG* (Rizzino, 2009).

Altogether, our data indicate that StemFlex is an efficient medium for the maintenance of human iPSCs. StemFlex-cultured cells retain characteristic iPSC morphology with round-shaped, tight morphology of colonies and expression of pluripotency markers. Little difference in global gene expression signatures between iPSCs cultured in StemFlex and TeSR-E8 could suggest that their formulations are highly similar, and also highlights the advantage of automation in stem cell research where eliminating human variability improves consistency and robustness of culture. Of note, automated passaging using ReLeSR was more efficient for cells maintained in StemFlex than in TeSR-E8. In our automation procedures, detaching cells from culture plates was faster with StemFlex than with TeSR-E8 and rarely required an additional “offline” step of “taping,” required for TeSR-E8. This was selected as an advantage of StemFlex for automation, for decreasing passaging time, reducing human intervention, and improving passaging efficiency. Our data also indicate that iPSCs cultured in both media exist in a metastable heterogeneous state supporting self-renewal, which is maintained by external cues within the culture media, in concordance with previous reports in mouse (Hanna et al., 2009, Najm et al., 2011) and human pluripotent stem cells (Buecker et al., 2010, Hough et al., 2014). These data also describe the single-cell resolution molecular pathways modulated in StemFlex and how they compare to those modulated in TeSR-E8.

### Limitations of Study

There are some limitations to this study. First, conducting the analysis at several time points would likely generate additional information regarding how signaling pathways of stem cells may be affected by prolonged culture in artificial *in vitro* conditions. The single time point used for the transcriptome analysis of single cells restricts the understanding of expression analysis over time. The repeat of such analysis at various passaging times could provide further information on the stability of cells in culture in both media. Furthermore, overall, the scRNA-seq dataset had relatively low number of mapped reads per cells: less than 20,000 unique molecular identifiers per cell (Figure S10). We addressed the limitation by utilizing the large number of cells (20,962 cells) and a stringent filtering step to remove cells with a low number of mapped reads and genes detected in a small percentage of cells. Another limitation is the small number of biological replicates used in the single-cell sequencing experiment, with two cell lines (from two genetic backgrounds) per medium. This could result in low detection power to reveal the effect derived from variation in genetic backgrounds from the scRNA-seq dataset. We complemented this deficit by using more cell lines, with 10 different genetic backgrounds for each medium, in the flow cytometry panel experiments. The cross-comparison between the two experimental approaches showed consistent results.

## Methods

All methods can be found in the accompanying Transparent Methods supplemental file.

## Data and Software Availability

We have made available both the raw and normalised data on ArrayExpress under accession E-MTAB-6524.

## Supplemental Information

Document S1. Transparent Methods and Figures S1–S10

Table S1. Reactome and DE Genes, Related to Figures 3, S5, and S6 Differential expression and functional enrichment analysis (Reactome differences in SF and E8; WAB0069 Reactome differences; WAB0450 Reactome differences; DE genes in SF and E8: 2 lines).

Table S2. Comparative Summary of Levels of Expression of Key Genes Involved in Pluripotency and Metabolism in StemFlex and TeSR-E8 Media (Pluripotency, Proliferation and Survival, Metabolism), Related to Figures 3, S5, and S6

Table S3. Pathway Analysis Subpopulations, Related to Figures 3 and S7–S9

Table S4. Comparing Subpopulations between Four Samples (Same Cell Line or Different Cell Lines) by a Machine Learning Classification Approach, Related to Figures 3 and S7–S9

Table S5. Percent Positive Cells by Subpopulations, Related to Figures 3 and S7–S9

Table S6. Pathway Enrichment and Gene Markers, Related to Figures 3 and S7–S9
