# Supplementary material for: Single-Cell Profiling Identifies Key Pathways Expressed by iPSCs Cultured in Different Commercial Media
Source: iScience. 2018 Aug 23;7:30–9. doi: 10.1016/j.isci.2018.08.016 (PMC6135898; doi:10.1016/j.isci.2018.08.016)
Supplement: Document S1. Transparent Methods and Figures S1–S10 [file mmc1.pdf]

ISCI, Volume 7

Supplemental Information

Single-Cell Profiling Identifies Key Pathways Expressed by iPSCs Cultured in Different Commercial Media

Maciej Daniszewski, Quan Nguyen, Hun S. Chy, Vikrant Singh, Duncan E. Crombie, Tejal Kulkarni, Helena H. Liang, Priyadharshini Sivakumaran, Grace E. Lidgerwood, Damián Hernández, Alison Conquest, Louise A. Rooney, Sophie Chevalier, Stacey B. Andersen, Anne Senabouth, James C. Vickers, David A. Mackey, Jamie E. Craig, Andrew L. Laslett, Alex W. Hewitt, Joseph E. Powell, and Alice Pébay

Supplementary Figures and Legends

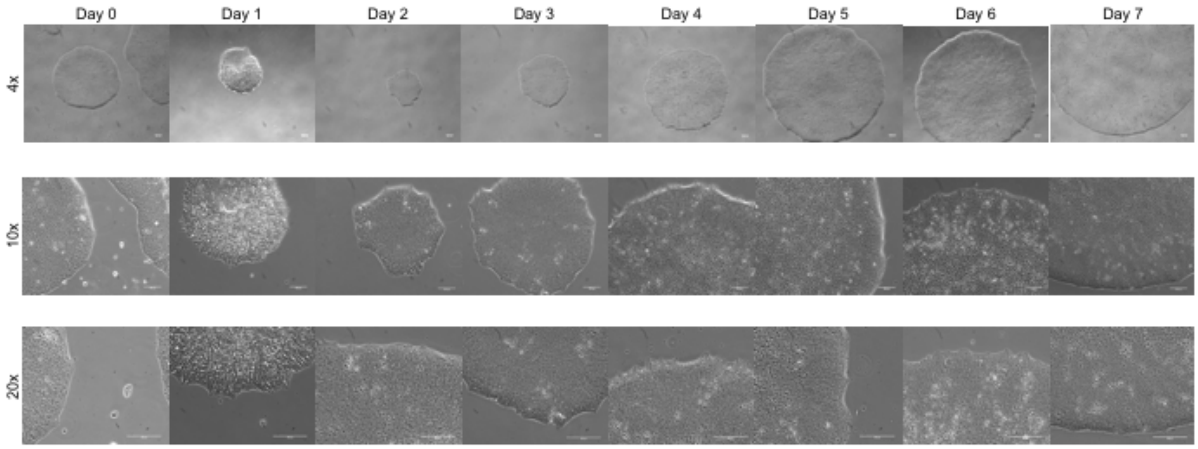

Figure S1: Representative morphology of iPSCs maintained in StemFlex^{TM}, Related to Figure 1. Images of a human iPSC line (MBE2906), from day 0 to day 7, at different magnifications (x4, 10 and 20). Cells were cultivated using an automated platform, on Vitronectin and in StemFlex^{TM}; medium was changed every 2-3 days. Images are representative of all cell lines. Scale bars: 100 μM.

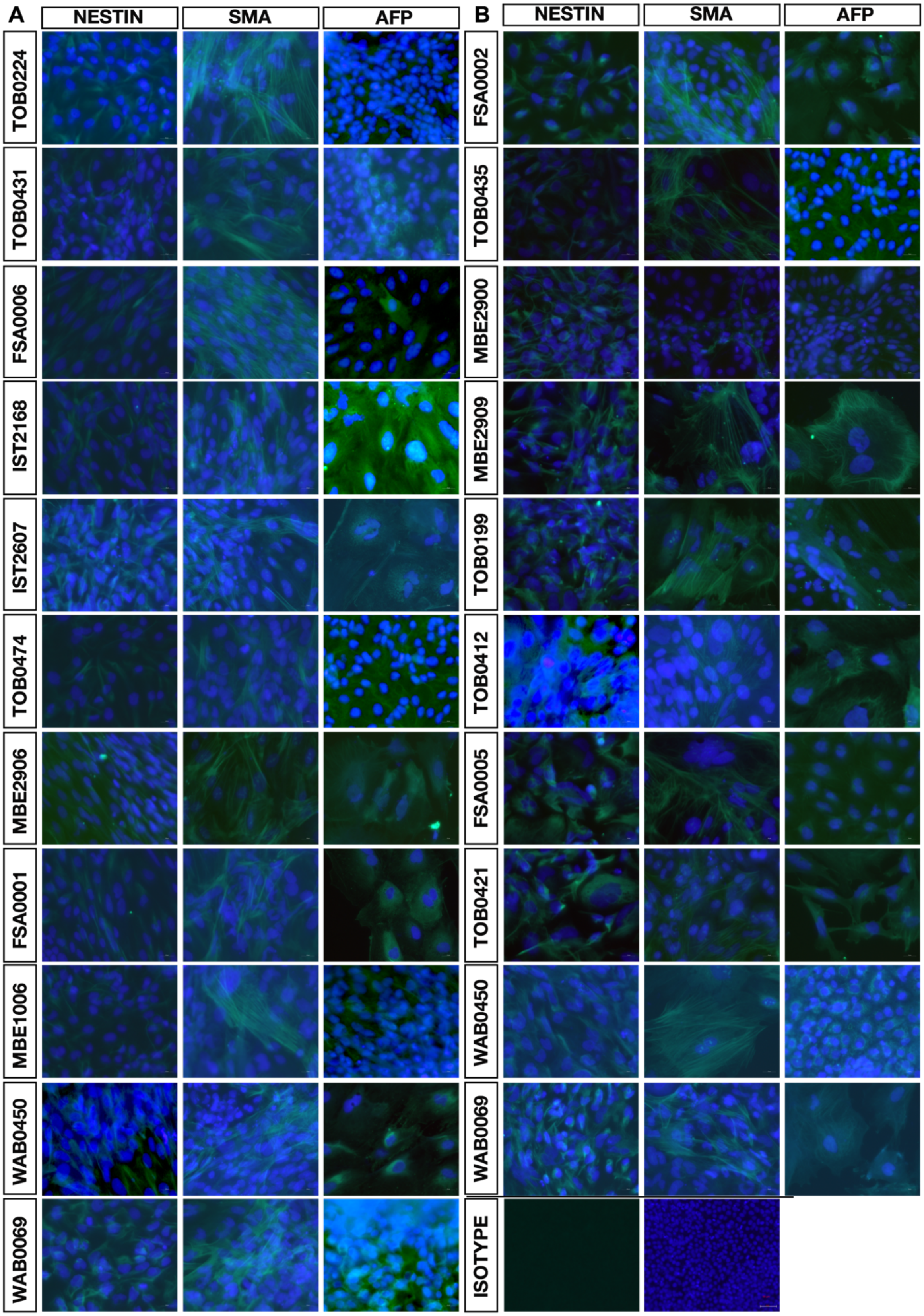

Figure S2: Differentiation of iPSCs into the three germ layers, Related to Figures 1, 2 and 3. Embryoid bodies of iPSCs maintained in (**A**) StemFlex™ and (**B**) TeSR™-E8™ showing differentiation into the three germ layers by immunostaining with NESTIN, SMA, AFP and with DAPI nucleic acid counterstain. Bottom right: representative negative isotype control with its corresponding DAPI. Scale bars: 20 μM.

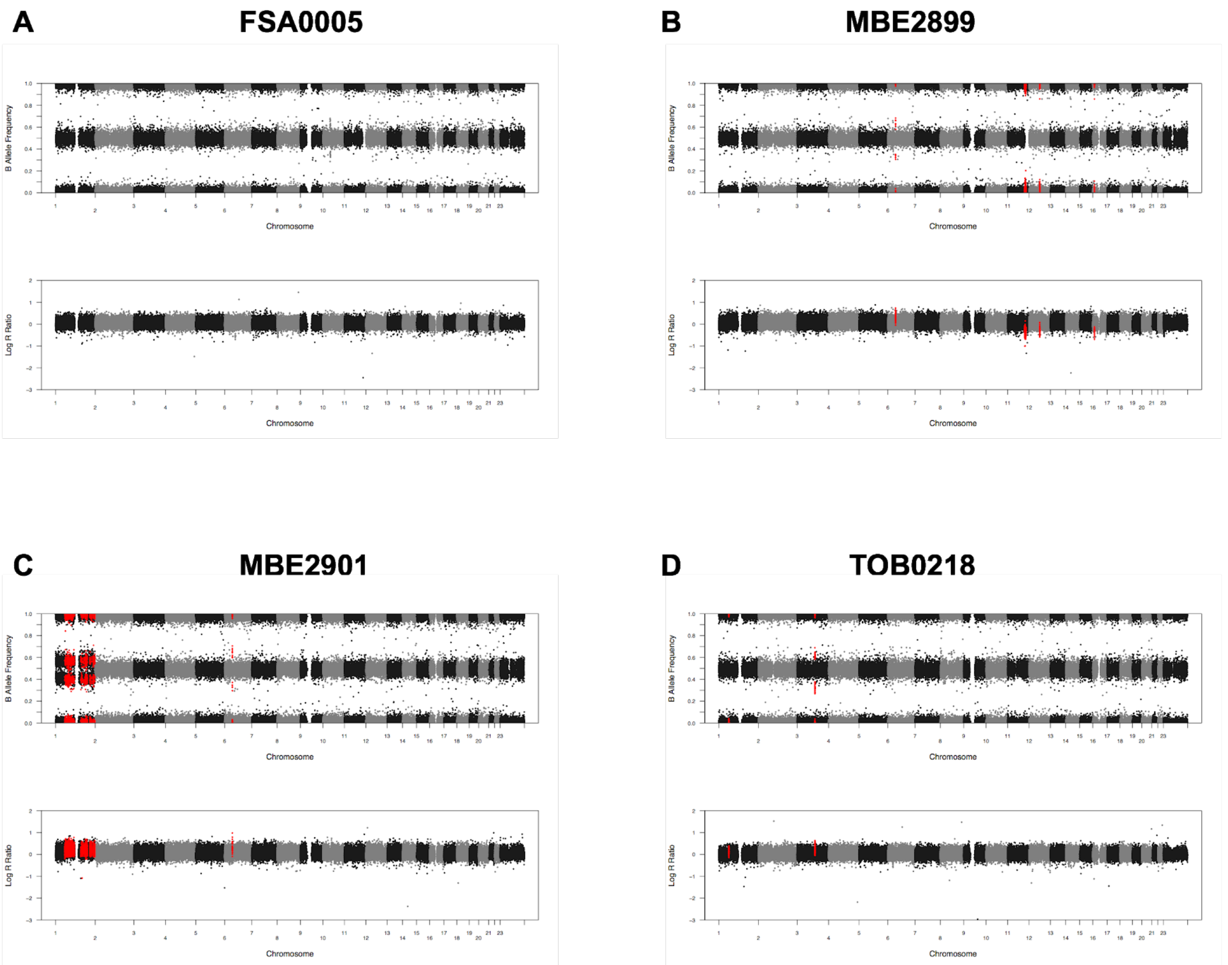

Figure S3: Copy Number Variation Analysis, Related to Figures 1 and 2. (**A**) Representative analysis of iPSCs with a normal virtual karyotyping (FSA0005). Anomalies were revealed in the iPSC lines MBE2899 (**B**), MBE2901 (**C**) and TOB0218 (**D**). Each panel shows the B allele frequency (BAF) and the log R ratio (LRR). BAF at values others then 0, 0.5 or 1 indicate an abnormal copy number. Similarly, the LRR represents a logged ratio of “observed probe intensity to expected intensity”. A deviation from zero corresponds to a change in copy number.

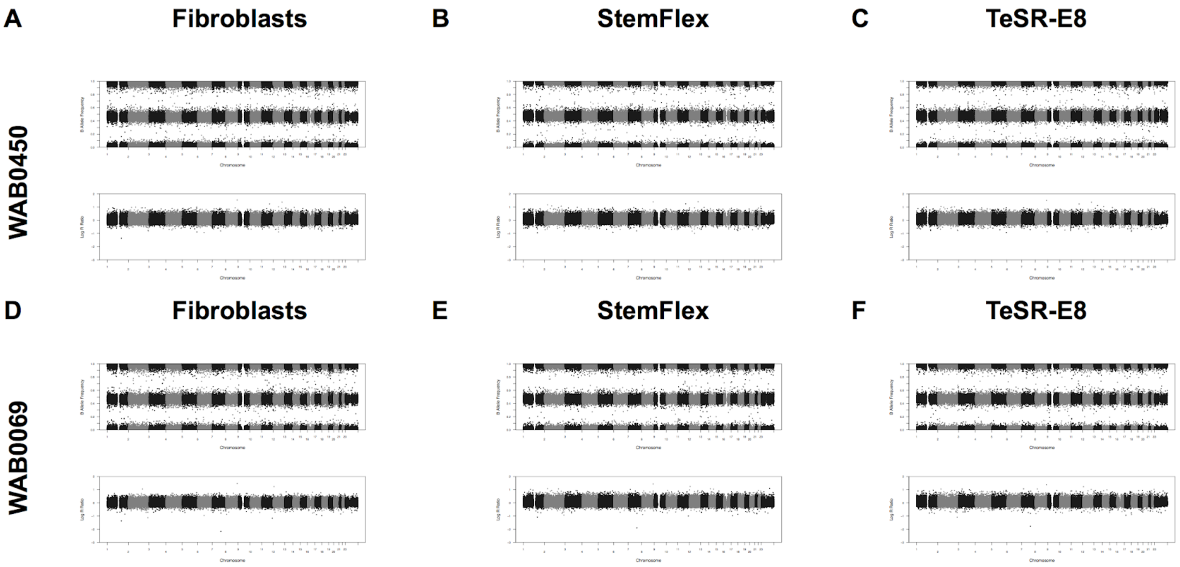

Figure S4: Characterisation of WAB0450 and WAB0069, Related to Figure 3. (**A-F**) Copy Number Variation Analysis of WAB0450 (**A-C**) and WAB0069 (**D-F**) in original fibroblasts (**A, D**), iPSCs at p8 in StemFlex™ (**B, E**) and TeSR™-E8™ (**C, F**). Each panel shows the B allele frequency (BAF) and the log R ratio (LRR).

A)

B)

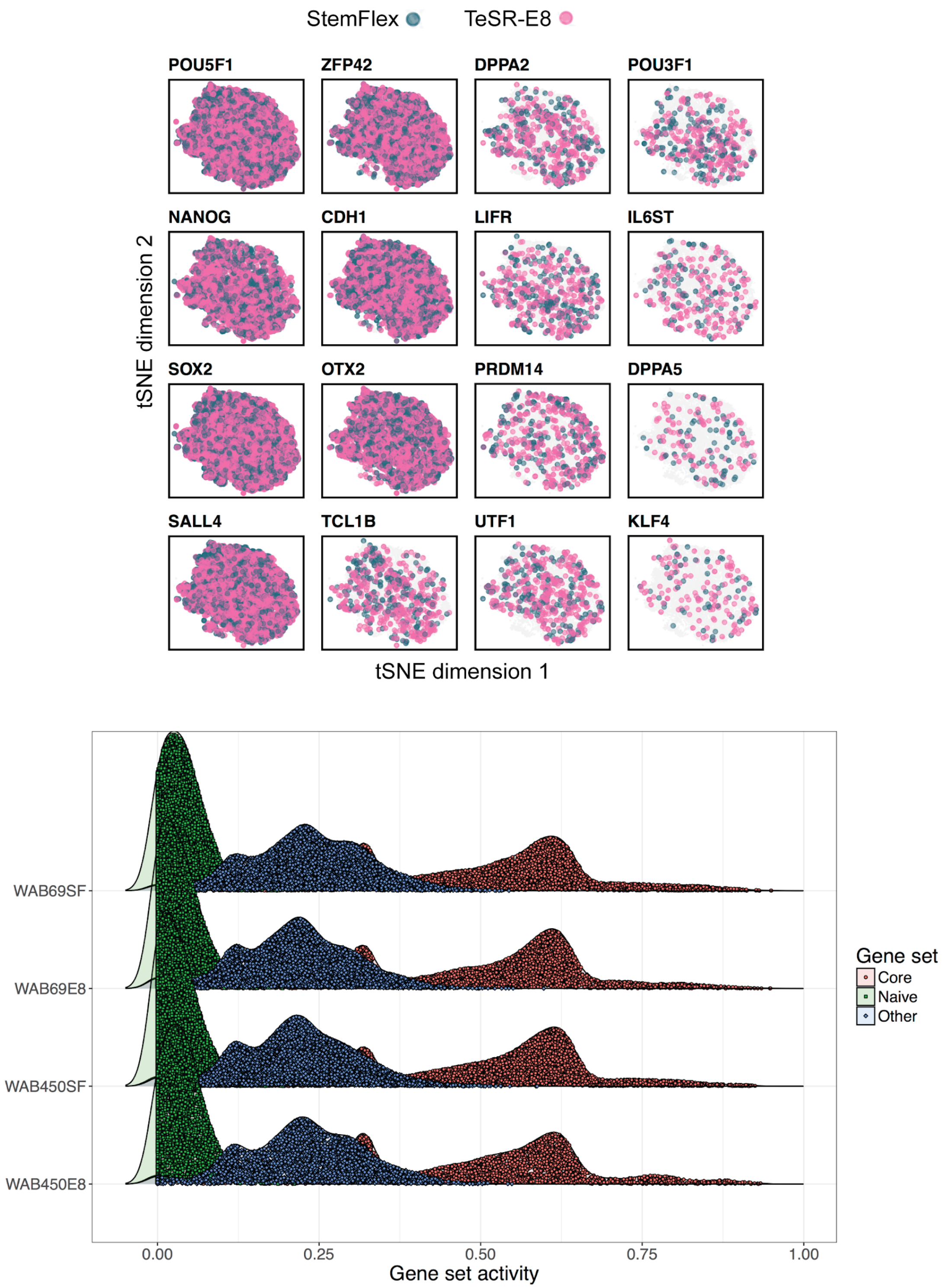

Figure S5. Expression of key pluripotency gene markers in the two media, Related to Figure 3 and Tables S1-2. (A) Cells in StemFlex™ (the pool of WAB0450SF and WAB0069SF cells) and TeSR™-E8™ (the pool of WAB0450E8 and WAB0069E8) expressing pluripotency markers are displayed in two dimensional tSNE plots. The majority of cells express common key pluripotency markers, such as *POU5F1* (*OCT4*), NANOG, *SOX2*, and *OTX2*. For all 16 genes shown in the figure, the number of expressing cells are even between the two media. (**B)** Gene set activities calculated by applying AUCell approach for three sets of known markers shown in Table S2. The regulation activity, ranging from 0 to 1, is the enrichment of the genes in the input gene set across the expression ranking of all genes in a cell. The three sets include "Core pluripotency markers" (Core), "Naïve pluripotency markers" (Naïve), and "Other pluripotency markers" (Other).

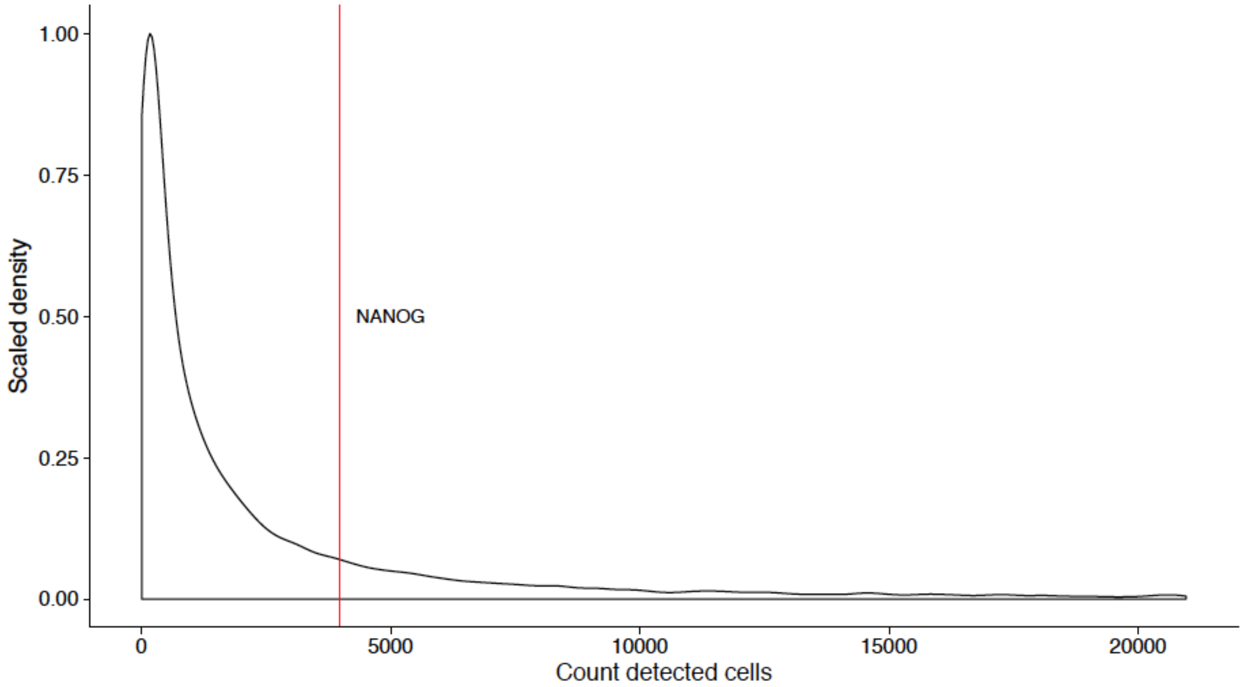

Figure S6. Distribution of *NANOG* in all cells, Related to Figure 3 and Tables S1-2. The percentage of cells expressing *NANOG* is at 75.5th percentile of all 16,270 reliably detected genes, higher than 13,349 genes.

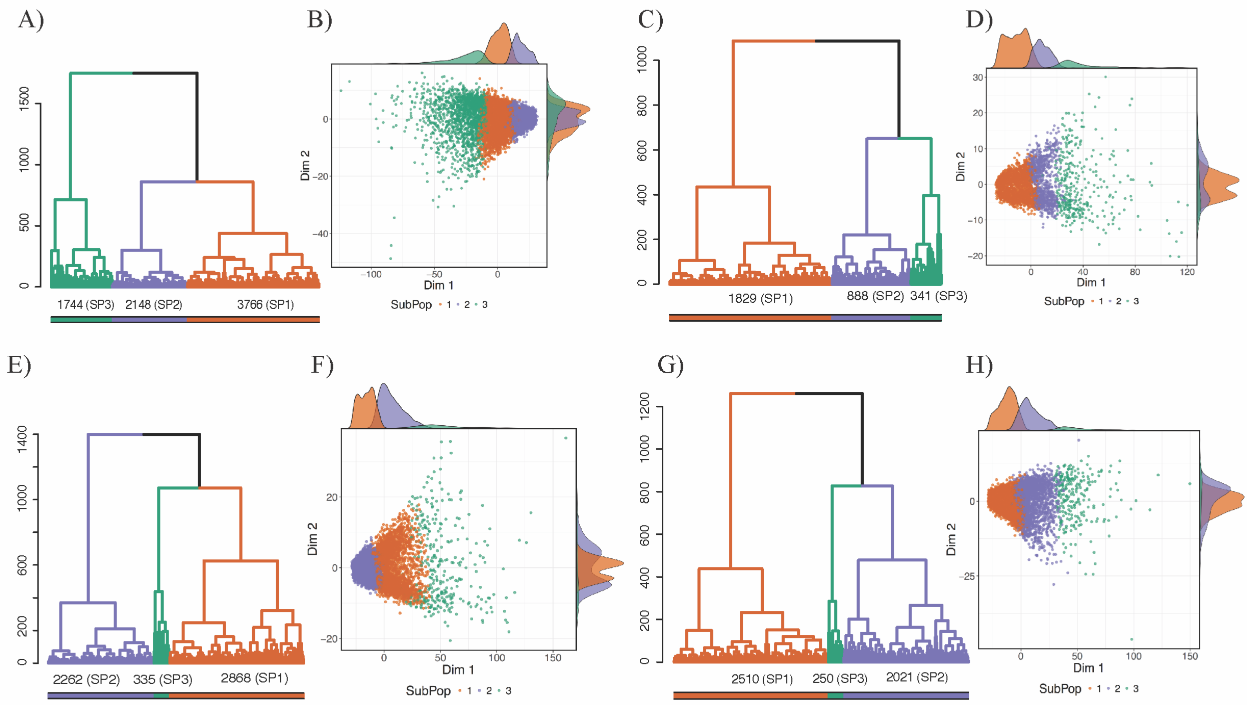

Figure S7: Single-cell subpopulation analysis of WAB0450 and WAB0069 in StemFlex™ and TeSR™-E8™ media, Related to Figure 3 and Tables S3-6. Subpopulations were identified by our unsupervised clustering algorithm so that a subpopulation consist of cells that are more similar to cells within the subpopulation compared to cells in other subpopulations. For each sample, a dendrogram tree with branches colored by subpopulations and the numbers of cells in each subpopulation are shown (panels **A**, **C**, **E**, and **G** for WAB0450SF, WAB0450E8, WAB0069SF and WAB0069E8, respectively). The distribution of cells in two first PCA principal components are shown as scatter plots accompanied by density plots on the right and top axes (panels **B**, **D**, **F**, and **H** for WAB0450SF, WAB0450E8, WAB0069SF and WAB0069E8 respectively).

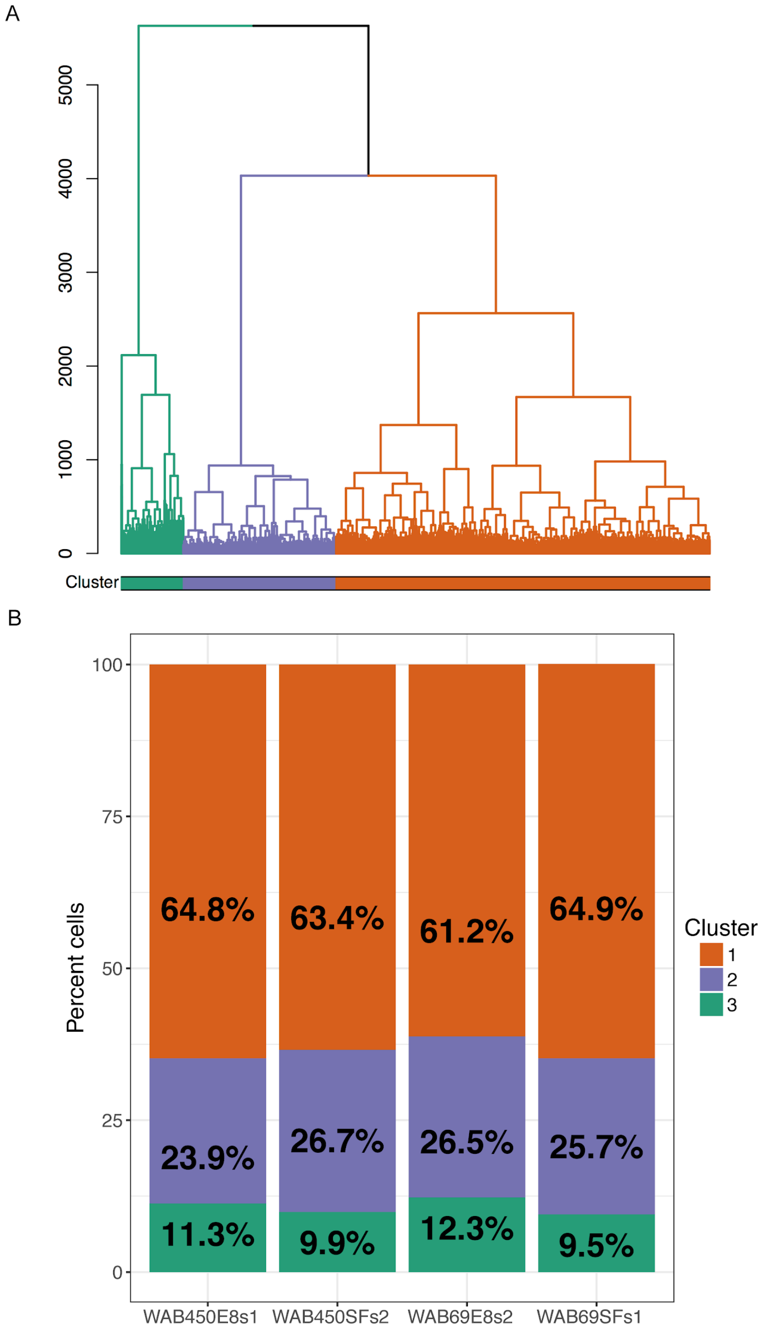

Figure S8. Subpopulation analysis of all cells in all four samples, Related to Figure 3 and Tables S3-6. (**A**) Dendrogram showing clustering results for more than 20,000 cells from the merged expression data of all four samples. The three clusters are shown by different colored branches. (**B**) The percent of cells distributed to each of the three clusters for each sample.

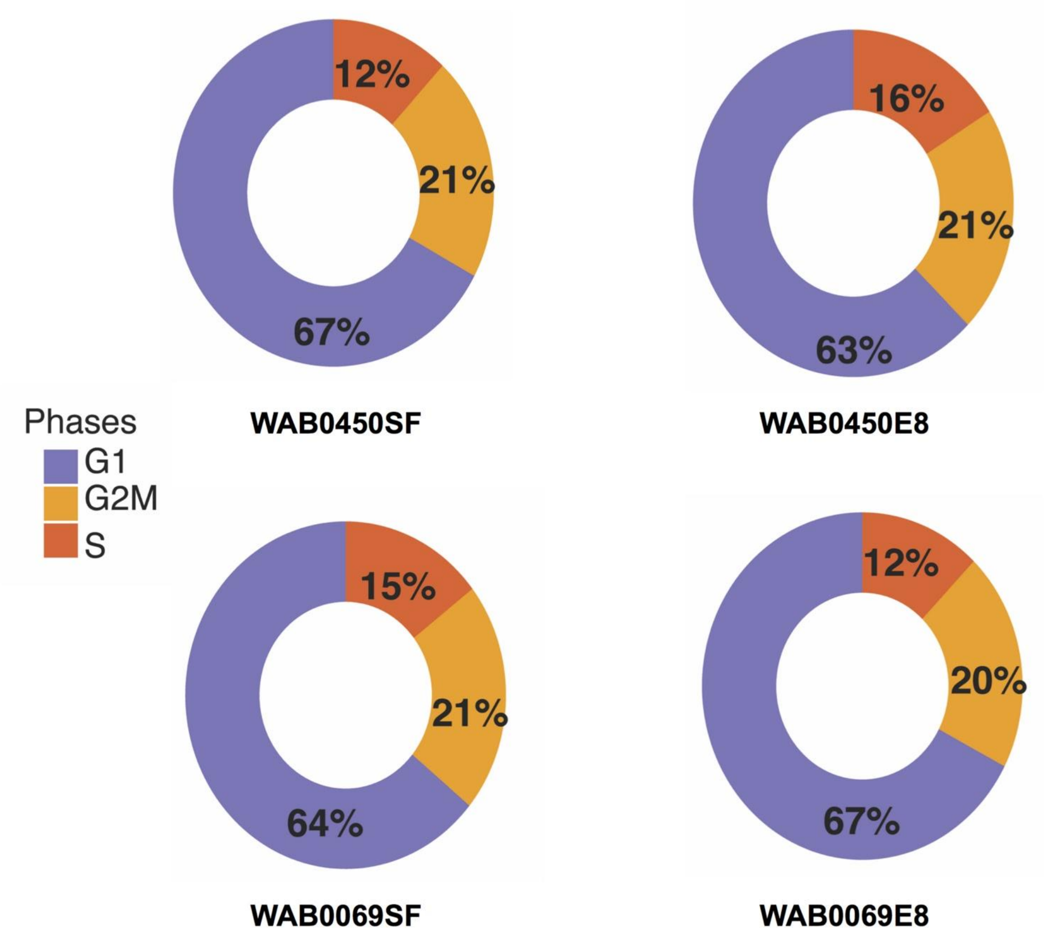

Figure S9. Predicted cell cycle stage for every cell in all combinations, Related to Figure 3 and Tables S3-6. WAB0450 and WAB0069 cells in StemFlex (SF) and TeSR-E8 (E8) showed no significant difference in proportions in the different cell cycle phases (G1, G2M, S).

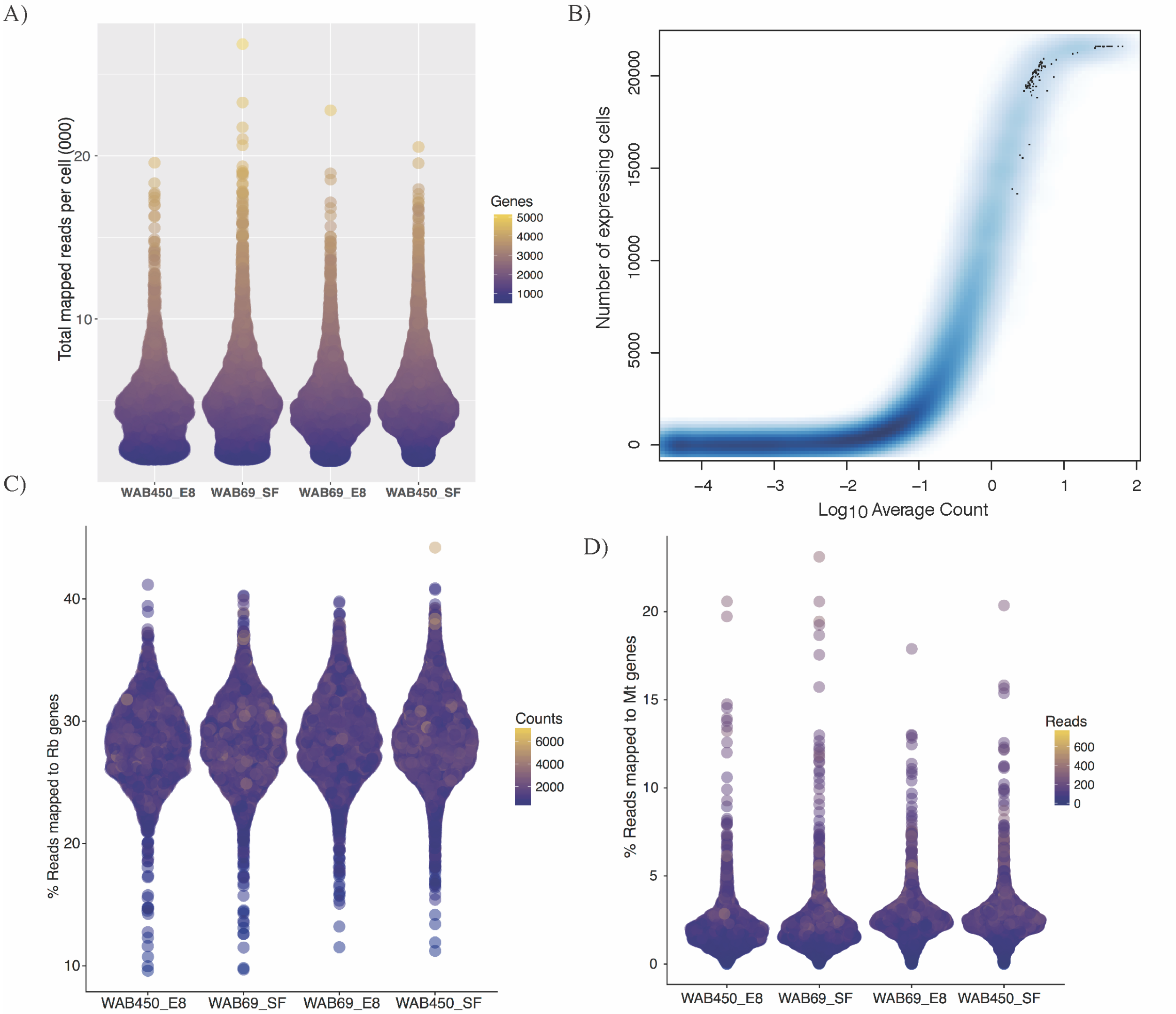

Figure S10: Quality control statistics for the single cell RNA sequencing of 21,597 human iPSCs, Related to Figure 3. (**A**)The library size (total mapped reads) range for each cell in each of the four samples. (**B**) Expression range of all genes detected. The range is greater than 6 orders of magnitudes. Genes detected in fewer than 0.1% of all cells were removed. (**C**) Percent reads mapped to mitochondrial genes as an indicator of cell conditions. The *x*-axis shows four samples, and the y-axis shows reads mapped. Cells with mitochondrial reads exceeding 3 x median absolute deviation (MAD) or mitochondrial reads above 20% were removed. (**D**) Percent reads mapped to ribosomal genes. We removed cells with over 3 x MAD or over 50% reads mapped to ribosomal genes.

Supplemental Tables added as Supplemental Data

Table S1. Reactome and DE genes. Differential expression and functional enrichment analysis (Reactome differences in SF and E8; WAB0069 reactome differences; WAB0450 reactome differences; DE genes in SF and E8_2lines), Related to Figures 3, S5 and S6.

Table S2. Comparative summary of levels of expression of key genes involved in pluripotency and metabolism in StemFlex™ and TeSR™-E8™ media (pluripotency; proliferation and survival; metabolism), Related to Figures 3, S5 and S6.

Table S3. Pathway analysis subpopulations, Related to Figures 3, S7-S9.

Table S4. Comparing subpopulations between four samples (same cell line or different cell lines) by a machine-learning classification approach, Related to Figures 3, S7-S9.

Table S5. Percent positive cells by subpopulations, Related to Figures 3, S7-S9.

Table S6. Pathway Enrichment & Gene Markers, Related to Figures 3, S7-S9.

Transparent Methods

Ethics. All experimental work performed in this study was approved by the Human Research Ethics committees of the Royal Victorian Eye and Ear Hospital (11/1031H) and University of Tasmania (H0014124) with the requirements of the National Health & Medical Research Council of Australia (NHMRC) and conformed with the Declarations of Helsinki (McCaughey et al., 2016).

Fibroblast Culture. Human skin biopsies were obtained from subjects over the age of 18 years. Fibroblasts were cultured in DMEM with high glucose, 10% fetal bovine serum (FBS), L-glutamine, 100 U/mL penicillin and 100 μg/mL streptomycin (all from Thermo Fisher Scientific, USA). All cell lines were mycoplasma-free (MycoAlert mycoplasma detection kit, Lonza, Switzerland). Fibroblasts at passage (p) 8-10 were used for reprogramming.

Generation, selection and maintenance of iPSCs. A TECAN liquid handling platform was used to cultivate cells, as described in (Crombie et al., 2017). iPSCs were generated by nucleofection (Amaxa™ Nucleofector™) with episomal vectors expressing *OCT-4*, *SOX2*, *KLF4*, *L-MYC*, *LIN28* and shRNA against *p53* (Okita et al., 2011) in feeder- and serum-free conditions using TeSR™-E7™ medium (Stem Cell Technologies, Canada) as described previously (Crombie et al., 2017). The reprogrammed cells were maintained on the automated platform using TeSR™-E7™ medium, with daily medium change. Pluripotent cells were selected by sorting anti-human TRA-1-60 Microbeads using a MultiMACS (Miltenyi, Germany) as described in (Crombie et al., 2017). Cell number was determined and cell were subsequently plated onto vitronectin XF™ (Stem Cell Technologies) in TeSR™-E8™ medium (Stem Cell Technologies) or StemFlex™ medium (Thermo Fisher Scientific). Subsequent culturing was performed on the automated platform using TeSR™-E8™ or StemFlex™ medium, and medium was changed every two days. Passaging was performed weekly on the automated platform using ReLeSR™ (Stem Cell Technologies) onto vitronectin XF™ coated plates as described in (Crombie et al., 2017).

iPSC quality control. TRA-1-60 quantifications were performed on a MACSQUANT immediately prior to passaging to fresh plates as described (Crombie et al., 2017). Pluripotency was assessed by expression of the markers OCT3/4 (sc-5279, Santa Cruz Biotechnology, USA), TRA-1-60 (MA1-023-PE, Thermo Fisher Scientific, USA). Copy number variation (CNV) analysis of original fibroblasts and iPSCs was performed using Illumina HumanCore Beadchip arrays. CNV analyses were performed using PennCNV (Colella et al., 2007) and QuantiSNP (Wang et al., 2007) with default parameter settings. Chromosomal aberrations were deemed to involve at least 20 contiguous single nucleotide polymorphisms (SNPs) or a genomic region spanning at least 1MB (Colella et al., 2007)(Wang et al., 2007). The B allele frequency (BAF) and the log R ratio (LRR) were extracted from GenomeStudio (Illumina, USA) for representation. Day 14 embryoid bodies (EBs) were plated on 24-well plates coated with 0.1% gelatin to differentiate for 7 days cultured in differentiation medium: DMEM, 20% FBS, 1% Non-Essential Amino Acid solution, 0.1mM β-mercaptoethanol and 1% Pen-strep. Germ layer differentiation was assessed by immunochemistry. For immunochemistry, cells were fixed with 4% paraformaldehyde, permeabilized with 0.1% Triton X-100 (Sigma-Aldrich) and blocked with 2% normal goat serum (Life Technologies). Standard immunostaining procedures were performed using the following primary antibodies: mouse anti- Nestin (Abcam, AB22035) for ectoderm; mouse anti- Smooth Muscle Actin (SMA, clone 1A4, R&D Systems, MAB1420) for mesoderm; mouse anti- Alpha-fetoprotein (AFP, Merck Millipore, ST1673) for endoderm. Secondary antibody used was Alexa Fluor 488 goat anti-mouse IgG (ThermoFisher, A11029). Nuclei were counterstained with DAPI (Sigma Aldrich).

Flow cytometry. The intracellular and extracellular immunolabelling of iPSCs and FACS analyses were all similarly performed as previously described (O'Brien et al., 2017). Briefly, cells grown in culture flasks or dishes were washed twice with 5 mL PBS -/- (Gibco, Life Technologies) and harvested using 1-2ml TrypLE Express (Gibco, Life Technologies) incubated for 5 minutes at 37°C. The cells were washed twice with PBS supplemented with 10% v/v FBS, which neutralises the TrypLE™ Express and help with cell viability before staining. The cells were resuspended in 10% v/v PBS -/- and aliquoted into individual 5ml FACs tubes for single extracellular labelling with primary monoclonal antibodies Anti-GPR64 (CSIRO, CSTEM7), Anti-CDCP1 (CSIRO, CSTEM26), Anti-F11R (CSIRO, CSTEM27), Anti-DSG2 (CSIRO, CSTEM28), Anti-CDH3 (CSIRO, CSTEM29), Anti-NLGN4X (CSIRO, CSTEM30), and Anti-PCDH1 (CSIRO, CSTEM31) and the stem cell markers TRA-1-60 and SSEA3 (both Merck Millipore). Primary and secondary antibodies were incubated for 20 minutes on ice and washed twice with PBS. The fluorescent secondary antibody goat anti-mouse IgG Alexa Fluor™ 488 (AF488, Thermo Fisher Scientific) was used to counterstain the primary antibodies. Non-cross reacted secondary antibodies were washed away with PBS and cells were resuspended with PBS containing 0.1% v/v propidium iodide (Sigma-Aldrich, USA). The propidium iodide was used to exclude non-viable cells during FACS analysis. For intracellular staining with Anti-OCT3/4 (Merck Millipore), cells were fixed with 4% paraformaldehyde for 30 minutes at RT, permeabilised with 0.01% Triton-X100 (Sigma-Aldrich) for 5 minutes and blocked with 10% Goat Serum (Thermo Fisher Scientific) for 30 minutes at RT after two washes with PBS. The fixed cells were labelled with Anti-OCT3/4 for 20 minutes at RT and washed twice with PBS. The secondary conjugated goat anti-mouse IgG1 AF488 fluorophore (Thermo Fisher Scientific) was used for the cross-reaction with Anti-OCT3/4 antibodies. The washed cells were finally resuspended in PBS for FACS analysis using BD™ Biosciences LSR II cell analyzer.

Single cell RNA sequencing. Cells were harvested using ReleSR™, colonies were dissociated into single cell suspension. Cells were counted and assessed for viability with Trypan Blue using a Countess II automated counter (Thermo Fisher Scientific), then pooled at a concentration of 391-663 cells/µL (3.91x10^5-6.63x10^5 cells/mL). Final cell viability estimates ranged between 85-97%. Cells were partitioned and barcoded using high-throughput droplet 10X Genomics Chromium™ Controller (10X Genomics, USA) and the Single Cell 3' Library and Gel Bead Kit (V2; 10X Genomics; PN-120237). The estimated number of cells in each well in the Chromium™ chip was optimized to capture approximately 8000-10,000 cells per cell pool. GEM generation and barcoding, cDNA amplification, and library construction were performed according to standard protocol. The resulting single cell transcriptome libraries were pooled and sequenced on an Illumina NextSeq™500, using a 150 cycle High Output reagent kit (NextSeq™500/550 v2; Illumina) in standalone mode as follows: 26bp (Read 1), 8bp (Index), and 98 bp (Read 2).

Bioinformatics mapping of reads to original genes and cells. Raw sequencing data (Illumina BCL files) were processed directly with the *cellranger* pipeline v2.0.0 (*mkfastq*, *count*, *aggr*) using the default parameters, except for the estimated cell number set at 5,000 cells. The reads were aligned to the *homo sapiens* GRCh38p10 Cell Ranger 2.0 reference genome using the STAR software (Dobin et al., 2013). Cell barcodes and unique molecular identifiers (UMI) were filtered using default parameters in the *cellranger count* processing. Reads with > 90% base calling accuracy were kept and UMI sequences were corrected based on high-quality sequenced UMIs with Hamming distance 1. A gene count expression matrix for four samples was generated using the *cellranger aggr* function.We have made available both the raw and normalised data on ArrayExpress under accession E-MTAB-6524.

Combining individual and cell SNP genotypes to assign cells to samples. We developed a new computational pipeline to combine SNP-chip data with scRNA SNP calling data in

individual cell to de-multiplex mixture of cells from different samples pooled into one sequencing reaction. Two cell lines, including WAB0450 and WAB0069 in TeSR^{TM}-E8^{TM} and StemFlex^{TM} media, were genotyped separately by Infinium HumanCore-24 v1.1 BeadChip assay (Illumina). GenomeStudio^{TM} V2.0 (Illumina) was used for SNP genotype calling of the BeadChip data. The full genotype report files were reformatted into Plink map, fam, and lgen files using custom Shell script and were then converted into variant calling format (vcf) using Plink 2 (Chang et al., 2015). For each sorted, indexed vcf file (separated by chromosomes), a strand fixing step was performed using bcf fixref function (Li, 2011). To increase the genome coverage of the BeadChip genotype data (total 306,670 SNPs), we performed imputation to the whole genome. Prior to imputation, Eagle V.2.3.5 was used for haplotype phasing the strand-fixed genotype vcf files (Loh et al., 2016). The phased data were imputed based on the 1000 genome phase 3 reference panel (2,535 samples) using the *minimac3* program (Fuchsberger, C Abecasis, GR Hinds, DA, n.d.). Single-cell SNPs were called from the mapped RNA BAM files using Freebayes V1.0.2 (Garrison and Marth, 2012). The likelihood that a cell originated from a sample is the cumulative likelihoods of variants identified in each cell. We applied Demuxlet software (Kang et al., 2017) to calculate posterior probability of a genotype *g* identified for a cell based on scRNA data given the DNA data from the imputed BeadChip genotypes. Each cell was assigned to the sample with the highest likelihood.

scRNA data filtering and normalisation. Sequencing reads from four samples were pooled and normalised at two levels - between samples and between cells. Briefly, to account for potential systematic measurement variation in total read depth per sample, we utilised unique information in read unique molecular identifiers (UMIs), sample indexes, and cell barcodes to perform random binomial sampling of reads. From all reads mapped to each gene in each cell, a subset of reads was randomly drawn from the binomial distribution, at the rate $Rate_i$, ($Reads_g$, $Rate_i$). $Rate_i$ was determined based on: total reads per sample, number of cells per sample, and fraction of mapped reads in a sample. This method has been shown to be less biased than other global-scaling methods and can equalise between sample sequencing depth while maintaining original read distribution (Zheng et al., 2017). The total mapped reads per cell may be affected by sequencing artefacts such as dropouts (RNA molecules not amplified at the initial PCR amplification round), PCR duplicates, or high amount of ribosomal RNA. Cells with high proportion of reads mapped to mitochondrial are likely cells suffering from more stress due to technical cell handling during library preparation and cell sorting. Transcriptional changes in those cells, therefore, do not reflect the biological changes of interest. After sample aggregation and between sample normalisation, cells and genes were filtered to remove potential technical noise in the data, ensuring subsequent analysis was not affected by cells that were inconsistently outside the threshold of three times the Median Absolute Deviation (MAD), which is a more robust method to detect outlier than using standard deviation around the mean (Leys et al., 2013). We calculated MAD for total mapped reads per cell, total genes detected in a cell, expression of mitochondrial, and expression of ribosomal genes for every cell in the data set (Figure S10). Using the outside 3 x MAD range, we found 163 cells with low or high total mapped reads, and 456 cells by high mitochondrial gene expression and 57 cells high ribosomal genes. After removing cells, we subsequently removed 16,459 genes that were detected in less than 0.1% of all remaining cells (Figure S9B). Following sample normalisation and cell-gene filtering, a pooling and deconvolution method was implemented to normalise variation in read depth between cells (Lun et al., 2016). The method sequentially and randomly pools 40, 60, 80 and 100 cells to form pseudo cells, in which the expression values of a gene across cells are summed. A size factor was calculated for each pool based on the summed expression values. The pool size factors then deconstructed into the size factors of individual cells by QR decomposition. This approach alleviates the statistical challenge in a dataset with an inflation of zero count measures,

including stochastic zero expression of genes that are lowly expressed (higher dropout rates), or genes that are turned on/off in different subpopulations of cells.

Differential expression and functional enrichment analysis. We compared gene expression for cells in TeSR™-E8™ and StemFlex™ media in three experimental designs: the pool of two cell lines, WAB0069 alone, and WAB0450 alone. General linear model and negative binomial test were implemented to identify differentially expressed (DE) genes as described in the DESeq package (Anders and Huber, 2010). In each condition, each cell was considered as one biological replicate. Prior to estimating dispersion and normalization factors, a pseudo count of one was added to the UMI count expression matrix, which was subtracted post DE test to adjust for estimated fold-change. Significantly differentiated genes were those with *p*-adjusted values less than 5% (Benjamini-Hochberg adjustment for false discovery rate). To identify functional pathways different between cell lines and media, we applied Reactome functional network analysis, a reliably curated functional network (Wu et al., 2014), to find enriched pathways in DE gene lists. We predicted cell cycle stage for every cell, and found no differences in cell cycle effects in all conditions (Figure S9).Therefore, we performed normalisation between cells and opted not to regress out cell cycle genes to maintain biological variation. To estimate the activity of three gene groups as listed on Table S2, we applied AUCell method (Aibar et al., 2017). The regulation activity, ranging from 0 to 1, is the enrichment of the genes in the input gene set across the expression ranking of all genes in a cell. The three sets include "core pluripotency markers", "Naïve pluripotency markers", and "Other pluripotency markers". A cell with a high gene set score suggests that the gene set actively regulate transcriptional state in the cell. As shown in Figure S5, no difference was observed in the activity of the three gene sets in the four samples, suggesting that the StemFlex and E8 equally maintain pluripotency states in the cell.

Statistical analysis. Data are expressed as mean ± standard error of the mean (SEM). All statistical analyses and graphical data were generated using Graphpad Prism software (v6, www.graphpad.com). Statistical methods utilised were two-tailed t-test and two-way ANOVA test followed by Sidak's multiple comparisons test. Statistical significance was established from $p<0.05$.

Supplemental References

Aibar, S., González-Blas, C.B., Moerman, T., Huynh-Thu, V.A., Imrichova, H., Hulselmans, G., Rambow, F., Marine, J.-C., Geurts, P., Aerts, J., van den Oord, J., Atak, Z.K., Wouters, J., Aerts, S., 2017. SCENIC: single-cell regulatory network inference and clustering. Nat. Methods 14, 1083–1086.

Anders, S., Huber, W., 2010. Differential expression analysis for sequence count data. Genome Biol. 11, R106.

Chang, C.C., Chow, C.C., Tellier, L.C., Vattikuti, S., Purcell, S.M., Lee, J.J., 2015. Second-generation PLINK: rising to the challenge of larger and richer datasets. Gigascience 4, 7.

Colella, S., Yau, C., Taylor, J.M., Mirza, G., Butler, H., Clouston, P., Bassett, A.S., Seller, A., Holmes, C.C., Ragoussis, J., 2007. QuantiSNP: an Objective Bayes Hidden-Markov Model to detect and accurately map copy number variation using SNP genotyping data. Nucleic Acids Res. 35, 2013–2025.

Crombie, D.E., Daniszewski, M., Liang, H.H., Kulkarni, T., Li, F., Lidgerwood, G.E., Conquest, A., Hernández, D., Hung, S.S., Gill, K.P., De Smit, E., Kearns, L.S., Clarke, L., Sluch, V.M., Chamling, X., Zack, D.J., Wong, R.C.B., Hewitt, A.W., Pébay, A., 2017. Development of a Modular Automated System for Maintenance and Differentiation of Adherent Human Pluripotent Stem Cells. SLAS Discov 2472555217696797.

Dobin, A., Davis, C.A., Schlesinger, F., Drenkow, J., Zaleski, C., Jha, S., Batut, P., Chaisson, M., Gingeras, T.R., 2013. STAR: ultrafast universal RNA-seq aligner. Bioinformatics 29, 15–21.

Fuchsberger, C Abecasis, GR Hinds, DA, n.d. minimac2: faster genotype imputation. Bioinformatics 31, 782–784.

Garrison, E., Marth, G., 2012. Haplotype-based variant detection from short-read sequencing. arXiv 1207.3907v2 [q–bio.GN].

Kang, H.M., Subramaniam, M., Targ, S., Nguyen, M., Maliskova, L., Wan, E., Wong, S., Byrnes, L., Lanata, C., Gate, R., Mostafavi, S., Marson, A., Zaitlen, N., Criswell, L.A., Ye, J., 2017. Multiplexing droplet-based single cell RNA-sequencing using natural genetic barcodes. https://doi.org/10.1101/118778

Leys, C., Ley, C., Klein, O., Bernard, P., Licata, L., 2013. Detecting outliers: Do not use standard deviation around the mean, use absolute deviation around the median. J. Exp. Soc. Psychol. 49, 764–766.

Li, H., 2011. A statistical framework for SNP calling, mutation discovery, association mapping and population genetical parameter estimation from sequencing data. Bioinformatics 27, 2987–2993.

Loh, P.-R., Danecek, P., Palamara, P.F., Fuchsberger, C., A Reshef, Y., K Finucane, H., Schoenherr, S., Forer, L., McCarthy, S., Abecasis, G.R., Durbin, R., L Price, A., 2016. Reference-based phasing using the Haplotype Reference Consortium panel. Nat. Genet. 48, 1443–1448.

Lun, A.T.L., Bach, K., Marioni, J.C., 2016. Pooling across cells to normalize single-cell RNA sequencing data with many zero counts. Genome Biol. 17, 75.

McCaughey, T., Liang, H.H., Chen, C., Fenwick, E., Rees, G., Wong, R.C.B., Vickers, J.C., Summers, M.J., MacGregor, C., Craig, J.E., Munsie, M., Pébay, A., Hewitt, A.W., 2016. An Interactive Multimedia Approach to Improving Informed Consent for Induced Pluripotent Stem Cell Research. Cell Stem Cell 18, 307–308.

O'Brien, C.M., Chy, H.S., Zhou, Q., Blumenfeld, S., Lambshead, J.W., Liu, X., Kie, J., Capaldo, B.D., Chung, T.-L., Adams, T.E., Phan, T., Bentley, J.D., McKinstry, W.J., Oliva, K.,

McMurrick, P.J., Wang, Y.-C., Rossello, F.J., Lindeman, G.J., Chen, D., Jarde, T., Clark, A.T., Abud, H.E., Visvader, J.E., Nefzger, C.M., Polo, J.M., Loring, J.F., Laslett, A.L., 2017. New Monoclonal Antibodies to Defined Cell Surface Proteins on Human Pluripotent Stem Cells. Stem Cells 35, 626–640.

Okita, K., Matsumura, Y., Sato, Y., Okada, A., Morizane, A., Okamoto, S., Hong, H., Nakagawa, M., Tanabe, K., Tezuka, K.-I., Shibata, T., Kunisada, T., Takahashi, M., Takahashi, J., Saji, H., Yamanaka, S., 2011. A more efficient method to generate integration-free human iPS cells. Nat. Methods 8, 409–412.

Wang, K., Li, M., Hadley, D., Liu, R., Glessner, J., Grant, S.F.A., Hakonarson, H., Bucan, M., 2007. PennCNV: an integrated hidden Markov model designed for high-resolution copy number variation detection in whole-genome SNP genotyping data. Genome Res. 17, 1665–1674.

Wu, G., Dawson, E., Duong, A., Haw, R., Stein, L., 2014. ReactomeFIViz: the Reactome FI Cytoscape app for pathway and network-based data analysis. F1000Res. https://doi.org/10.12688/f1000research.4431.1

Zheng, G.X.Y., Terry, J.M., Belgrader, P., Ryvkin, P., Bent, Z.W., Wilson, R., Ziraldo, S.B., Wheeler, T.D., McDermott, G.P., Zhu, J., Gregory, M.T., Shuga, J., Montesclaros, L., Underwood, J.G., Masquelier, D.A., Nishimura, S.Y., Schnall-Levin, M., Wyatt, P.W., Hindson, C.M., Bharadwaj, R., Wong, A., Ness, K.D., Beppu, L.W., Deeg, H.J., McFarland, C., Loeb, K.R., Valente, W.J., Ericson, N.G., Stevens, E.A., Radich, J.P., Mikkelsen, T.S., Hindson, B.J., Bielas, J.H., 2017. Massively parallel digital transcriptional profiling of single cells. Nat. Commun. 8, 14049.
